# Roles of visual and non-visual information in the perception of scene-relative object motion during walking

**DOI:** 10.1167/jov.20.10.15

**Published:** 2020-10-14

**Authors:** Mingyang Xie, Diederick C. Niehorster, Markus Lappe, Li Li

**Affiliations:** School of Psychology and Cognitive Science, East China Normal University, Shanghai, China; New York University-East China Normal University Institute of Brain and Cognitive Science at New York University Shanghai, Shanghai, China; Institute for Psychology, University of Muenster, Muenster, Germany; Institute for Psychology, University of Muenster, Muenster, Germany; School of Psychology and Cognitive Science, East China Normal University, Shanghai, China; New York University-East China Normal University Institute of Brain and Cognitive Science at New York University Shanghai, Shanghai, China; Faculty of Arts and Science, New York University Shanghai, Shanghai, China

**Keywords:** self-movement, optic flow, flow parsing, object motion, multi-sensory information

## Abstract

Perceiving object motion during self-movement is an essential ability of humans. Previous studies have reported that the visual system can use both visual information (such as optic flow) and non-visual information (such as vestibular, somatosensory, and proprioceptive information) to identify and globally subtract the retinal motion component due to self-movement to recover scene-relative object motion. In this study, we used a motion-nulling method to directly measure and quantify the contribution of visual and non-visual information to the perception of scene-relative object motion during walking. We found that about 50% of the retinal motion component of the probe due to translational self-movement was removed with non-visual information alone and about 80% with visual information alone. With combined visual and non-visual information, the self-movement component was removed almost completely. Although non-visual information played an important role in the removal of self-movement-induced retinal motion, it was associated with decreased precision of probe motion estimates. We conclude that neither non-visual nor visual information alone is sufficient for the accurate perception of scene-relative object motion during walking, which instead requires the integration of both sources of information.

## Introduction

Accurate and precise estimation of scene-relative object motion is important during self-movement. When one moves through the world, the projected image of objects in the scene forms a moving pattern on the eye, which is termed “optic flow” (Gibson, 1950; Gibson, 1958). If an object moves in the scene during one's self-movement, its retinal motion is due to both scene-relative object motion and one's self-movement in the world. To estimate scene-relative object motion accurately, the visual system must remove the self-movement component from the retinal motion of the object.

Warren and Rushton (2007, 2008, 2009; see also Rushton & Warren, 2005) proposed that the visual system can parse out the self-movement component and attribute the remaining retinal motion to scene-relative object movement. They conducted a series of experiments and found that optic flow plays an important role for the visual system to identify and parse out the self-movement component and thus termed this process “flow parsing.” However, parsing out the self-movement component is incomplete based on visual information (such as optic flow) alone (e.g., Matsumiya & Ando, 2009; Niehorster & Li, 2017; Swanston & Wade, 1988; Wexler, 2003). Non-visual information (such as vestibular, somatosensory, and proprioceptive information) generated during self-movement has been reported to contribute to the identification of scene-relative object motion during self-movement. For example, Dyde and Harris (2008) found that self-movement in the form of sinusoidal oscillation affected the extent to which a fixated object had to move in space to appear earth stationary to the observer. MacNeilage, Zhang, DeAngelis, and Angelaki (2012) asked observers to discriminate object motion during simulated self-movement through a star-field scene with or without scene-consistent vestibular stimulation. They found that observers had a better performance with added vestibular information than with visual information alone. Likewise, Dokka, MacNeilage, DeAngelis, and Angelaki (2015) asked observers to judge object motion trajectory and found a larger compensation for the self-movement component with combined visual and vestibular information than with visual information alone. Dupin and Wexler (2013) asked observers to judge the speed of object rotation and found similar results even though they placed visual and non-visual information in conflict. Finally, Fajen and his colleagues (Fajen & Matthis, 2013; Fajen, Parade, & Matthis, 2013) had participants walk to avoid moving obstacles in a virtual environment and found that participants relied on both visual and non-visual information about self-movement to judge whether they could safely pass.

**Figure 1.**
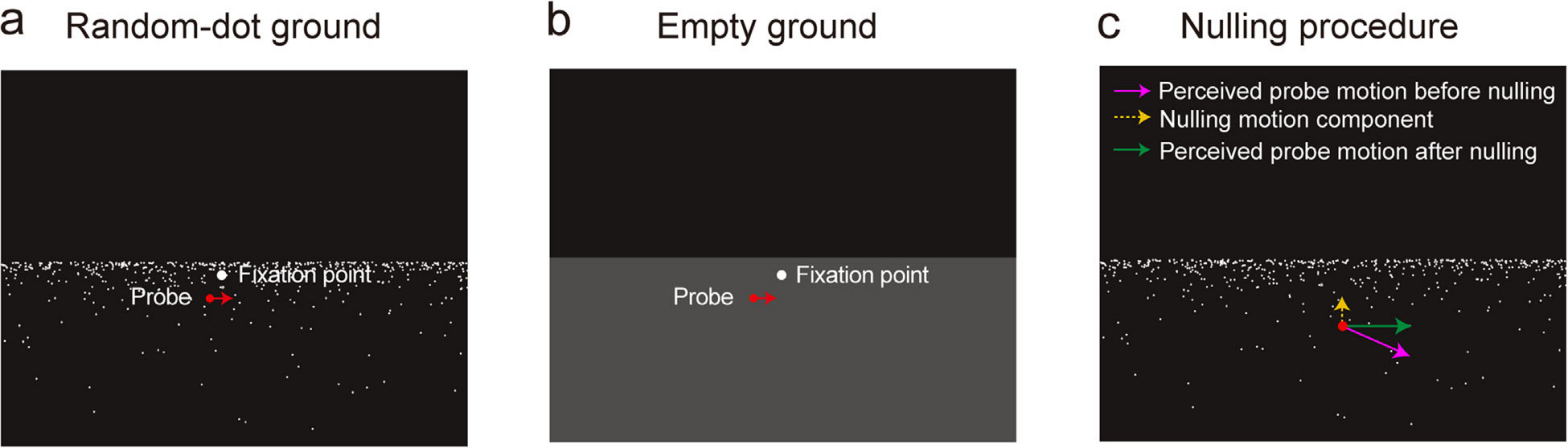
Illustrations of the visual stimulus types. (a) A random-dot ground scene. (b) An empty ground scene. The illustrated probe motion in the scene is rightward in both scenes. (c) A conceptual illustration showing the motion components in the nulling procedure.

Despite the above findings, to the best of our knowledge no study so far has quantitatively and systematically examined the contribution of visual versus non-visual information to the estimation of scene-relative object motion during walking, a common form of self-movement in daily life. Accordingly, it still remains in question how accurately people perceive scene-relative object motion during walking using visual information alone, non-visual information alone, or combined visual and non-visual information. In this study, we aimed to address this question. We placed a probe object on the ground that moved in the scene during simulated or real walking and used a nulling method developed by Niehorster and Li (2017) to directly measure to what extent the retinal motion component of the probe due to self-movement could be removed to determine the accuracy of the estimation of scene-relative object motion during walking. We tested three stimulus conditions: (1) In the visual-information condition, participants stood still and passively viewed through a head-mounted display (HMD) the scene that simulated smooth forward walking over a random-dot ground plane with no head bobbing and body sway (Figure 1a and Supplementary Movie S1). Participants were thus provided with noise-free visual information (such as optic flow from the random-dot ground plane) about translational self-movement in this condition. (2) In the non-visual-information condition, participants walked straight in the real world while viewing through the HMD the online-generated scene that corresponded to their walking straight over an empty ground (Figure 1b and Supplementary Movie S2) in a virtual environment. The empty ground provided no optic flow and effectively eliminated any visual information about translational self-movement. Participants were thus provided with mainly non-visual information about self-movement (such as vestibular, somatosensory, and proprioceptive information) generated from walking in this condition. (3) In the combined-information condition, participants walked straight while viewing through the HMD the online-generated scene that corresponded to their walking over a random-dot ground in a virtual environment that generated optic flow (Supplementary Movie S3). Participants were thus provided with combined visual and non-visual information about self-movement in this condition. By measuring the proportion of translational self-movement component subtracted from the retinal motion of the probe in these three conditions, we systematically examined the accuracy and the precision of the estimation of scene-relative object motion during walking based on visual information alone, non-visual information alone, or combined visual and non-visual information.

## Experiment 1: Estimation of scene-relative object motion during walking

The logic of this experiment is as follows. If non-visual information contributes to the accurate perception of object motion during walking, then the translational self-movement component subtracted from the retinal motion of the probe should be larger than zero in the non-visual-information condition. If visual information such as optic flow plays a more important role than does non-visual information, a larger translational self-movement component should be subtracted from the retinal motion of the probe in the visual than in the non-visual condition. Due to the fact that we have access to both visual and non-visual information during walking in the natural world, when both visual and non-visual information about self-movement is available in the combined condition, we expect the most complete subtraction of translational self-movement component from the retinal motion of the probe that would lead to the most accurate and precise estimation of scene-relative object motion during walking.

### Methods

#### Participants

Twelve participants (all naive as to the specific goals of the study; three males, nine females) between the ages of 21 and 26 years participated in this experiment. All had normal or corrected-to-normal vision and provided informed consent. The study was approved by the Institutional Review Board at the New York University Shanghai.

#### Visual stimuli and apparatus

The stimulus depicted an empty gray ground (luminance contrast, 67%; maximum depth, 100 m) (Figure 1b) or a random-dot ground consisting of 1000 non-expanding white dots (luminance contrast, 96%) randomly distributed on the ground such that about 500 dots were visible in the viewing frustum (i.e., on the screen) in each frame (Figure 1a). The same number of dots was placed in equal intervals in depth to ensure the presence of a sufficient number of dots in the foreground.

We tested three stimulus conditions: (1) In the visual-information condition, participants stood still and viewed a scene that simulated smooth linear forward self-movement (i.e., with no head bobbing and body sway) at the average walking speed of 1 m/s over the random-dot ground through an Oculus DK2 HMD (diagonal field of view, 100°; resolution, 1080H × 960V pixels; Oculus, Menlo Park, CA) in stereo mode (Figure 1a and Supplementary Movie S1). This condition provided participants with noise-free visual information about translational self-movement (such as optic flow of the random-dot ground). (2) In the non-visual-information condition, participants were instructed to walk straight at their normal walking speed while viewing the empty ground scene (Figure 1b and Supplementary Movie S2) through the HMD. The head orientation and position were tracked by an optical tracking system (WorldViz PPT-N, 180 Hz; WorldViz, Inc., Santa Clara, CA) and were used to update the scene to mimic the participant walking straight over an empty ground in a virtual environment. The empty ground provided no optic flow and effectively eliminated any visual information about translational self-movement. This condition thus provided participants with mainly non-visual information such as vestibular, somatosensory, and proprioceptive information about self-movement during walking. (3) In the combined-information condition, participants were instructed to walk straight while viewing the random-dot ground scene through the HMD (Supplementary Movie S3). This condition thus provided participants with both visual and non-visual information about translational self-movement.

In all three stimulus conditions, at the beginning of each trial, a blue non-expanding fixation point (1° diameter) was placed on the ground along the *z*-axis at 10 m away from the observer. The fixation point moved with the observer movement, always keeping 10 m distance away from the observer, such that the gaze angle of the observer was kept constant and no pursuit eye movement was induced throughout the trial. In the visual-information condition, participants were instructed to fixate the fixation point and watched the simulation of smooth translational self-movement along the *z*-axis of the virtual world. The blue fixation point turned into green after 1 second. In the other two stimulus conditions, participants were instructed to fixate the blue fixation point and walk straight toward it at 1 m/s. When participants reached the target speed of 1 ± 0.1 m/s, the blue fixation point turned into green. If the participant's walking speed exceeded 1.1 m/s, the color of the fixation point would turn into red. When the green fixation point was displayed for 1 second (i.e., the participant maintained a straight walking speed of 1 m/s for 1 second in the two walking conditions), the fixation point disappeared, and 150 ms later a red probe dot (1° diameter) appeared and moved sideways over the ground perpendicular to the observer's axis of translation at a retinal angular speed of 6°/s for 500 ms while the participant continued to approach it at the same walking speed. The midpoint of the trajectory of the probe was positioned right in front of the participant along the *z*-axis at an angular declination (*θ*) of 15° below the horizon. The distance of the probe in the virtual world (*Z*) depends on the participant's eye height, as given by

(1) Z=eyeHeight/tanθ

In order to find the correct nulling motion component, we calculated the retinal motion component of the probe due to translational self-movement along the *z*-axis (*v_self_*) when the probe was at the midpoint of its trajectory right in front of the observer, which is given by

$$v_{self}=T_{z}\sin2\theta /\left(2Z\right)$$

in which *T_z_* is the speed of the translational self-movement. This formula can be rewritten as

(2) $$\;v_{self}=\frac{T_{z}\sin\left(2\theta \right)\tan\theta }{2eyeHeight}=T_{z}\sin^{2}\left(\theta \right)/eyeHeight$$

On each trial, a nulling motion component $\left(T_{z}^{''}\right)$ along the *z*-axis of the virtual world was added to the motion of the probe on the ground (Figure 1c) by a Bayesian adaptive method (Kontsevich & Tyler, 1999). In retinal coordinates, this nulling motion component (*v_n_*) is given by

(3) $$v_{n}=T_{z}^{''}\sin^{2}\left(\theta \right)/eyeHeight$$

At the end of each trial, participants were asked to report whether the probe approached or receded over the ground plane in the virtual world using a handheld controller. The visual size of the probe scaled naturally with its original motion and the added nulling motion in the virtual world. If, during walking, participants did not manage to maintain an average speed of 1 ± 0.1 m/s for 1 second, then the trial was aborted and the participant was asked to return to the starting position, upon which the trial was restarted.

We used a fixation point to control eye movements to remove any compensation for self-movement associated with eye movements (Blohm, Missal, & Lefevre, 2003; Blohm, Missal, & Lefevre, 2005; Blohm, Optican, & Lefevre, 2006; Brenner, Smeets, & Van den Berg, 2001; Daye Blohm, & Lefèvre, 2010; Dessing, Crawford, & Medendorp, 2011; Van Pelt & Medendorp, 2008). The fixation point was placed on the ground instead of on the horizon. This is because the probe had to be close to the moving observer to have a significant self-movement component, and was thus placed at an angular declination of 15° below the horizon. Accordingly, if the fixation point were placed on the horizon, the moving probe target would have become too eccentric for a participant to perceive its motion well.

All visual stimuli were presented through the HMD in stereo mode. All were generated on a Dell (Round Rock, TX) computer with a 4.0 GHz Core i7 processor (Intel Corp., Santa Clara, CA) and Nvidia (Round Rock, TX) GeForce GTX TITAN X graphics card at a frame rate of 60 Hz running Windows 8.1 Pro (Microsoft, Redmond, WA), and were programmed in Vizard 5 (WorldViz). The experiment was conducted in a room 12 m × 9 m.

#### Procedure

Before the start of each trial, participants went through a calibration procedure; that is, the participant saw the top view of the experimental setup with two arrows: a yellow arrow and a red arrow. Each participant was then asked to move his/her body (represented by the yellow arrow), which was tracked by the optical tracking system to align it with the start position (represented by the red arrow). When the arrows were aligned, an egocentric view of the virtual world depicting a white point overlaid by two green triangles that were placed symmetrically in the center of the display was shown for fine adjustment of the participant's facing direction and body orientation. Each participant was instructed to rotate his/her body and head to put the white point in between the two green triangles to ensure that at the start of each trial the participant's body and head were oriented to face the *z*-axis of the virtual world. After participants held that position for 1 second, the trial started, which was indicated by a sound and the disappearance of the white dot and green triangles.

The experiment consisted of three blocks of 40 trials with each block corresponding to one of the three stimulus conditions. The testing order of the stimulus conditions was counterbalanced between participants. Before each block, participants received five practice trials from the stimulus condition they were about to perform. The experiment lasted about 1 hour.

#### Data analysis

Figure 1c illustrates the concepts behind the nulling procedure. The magenta arrow indicates the perceived scene-relative probe motion after the visual system performs flow parsing—that is, using optic flow to partially subtract the translational self-movement component from the retinal motion of the probe (e.g., Matsumiya & Ando, 2009; Niehorster & Li, 2017). When flow parsing is partial, the incomplete removal of the self-movement component leads to the perception of probe motion in the scene toward the participant (i.e., the downward motion in the magenta arrow). The residual retinal motion of the probe due to self-movement can be nulled by adding a motion component (depicted by the yellow dotted arrow) under the control of a Bayesian adaptive method that allows for acquisition of both the threshold and slope of the psychometric function (Kontsevich & Tyler, 1999). This method thus identifies both the point of subjective equality (PSE) corresponding to the magnitude of the added nulling motion component when the probe is perceived to neither approach nor recede from the participant in the virtual world (depicted by the green arrow) and the standard deviation (*σ*) of the cumulative Gaussian fit to the psychometric function that quantifies the precision of probe motion estimates.

As in Niehorster and Li (2017), we used the retinal motion component of the probe due to translational self-movement (*v_self_*) and the PSE of the nulling motion component in retinal coordinates (*v_n_*) to calculate the percentage compensation gain, the extent to which the visual system could remove the retinal motion component of the probe due to self-movement to recover the probe's scene-relative motion, which is given by

(4) $$\%\;\mathrm{Gain}=\left(1-\frac{v_{n}}{v_{self}}\right)*100\%$$

### Results and discussion

**Figure 2.**
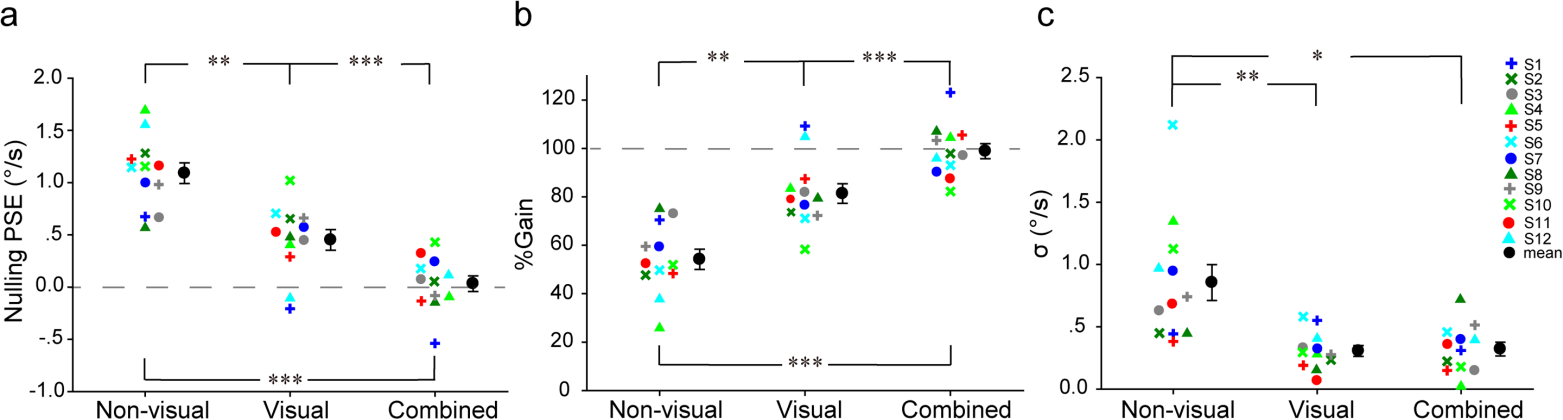
Experiment 1 data. (a) Nulling PSE, (b) percentage compensation gain, and (c) standard deviation (*σ*) of the cumulative Gaussian fit for each participant along with the mean against the three stimulus conditions. Error bars are ±1 *SE* across 12 participants. ^*^p < 0.05, ^**^p < 0.01, and ^***^p < 0.001.

Figure 2a plots the nulling PSE against the three stimulus conditions for each participant along with the mean. A one-way repeated-measures ANOVA on the nulling PSE revealed a significant effect of stimulus condition, *F*(2, 22) = 48.29, *p* < 0.001, *η*^2^ = 0.81. Bonferroni tests revealed that the mean nulling PSE for the non-visual condition (mean ± *SE*, 1.10 ± 0.099) was significantly larger than that for the visual condition (0.45 ± 0.098, *p* = 0.0020) and the combined condition (0.032 ± 0.074, *p* < 0.001). The mean nulling PSE for the visual condition was also significantly larger than that for the combined condition (*p* < 0.001).

Figure 2b plots the percentage compensation gain against the three stimulus conditions for each participant along with the mean. As expected, the larger the nulling PSE, the smaller the percentage compensation gain. Due to the fact that the percentage compensation gain is a linear transformation of the nulling PSE, as expected, the mean percentage compensation gain for the non-visual condition (54% ± 4%) was significantly smaller than that for the visual condition (81% ± 4%) and the combined condition (98% ± 3%). The mean percentage compensation gain for the visual condition was also significantly smaller than that for the combined condition. A separate one-sample *t*-test revealed that the mean percentage compensation gain for the combined condition was not significantly different from 100%, *t*(11) = –0.35, *p* = 0.73).

Figure 2c plots the standard deviation (*σ*) of the cumulative Gaussian fit to the psychometric function against the three stimulus conditions for each participant along with the mean. A one-way repeated-measures ANOVA on the *σ* revealed a significant effect of stimulus condition, *F*(2,22) = 12.91, *p* = 0.0020, *η*^2^ = 0.54). Bonferroni tests revealed that the mean *σ* for the non-visual condition (0.86 ± 0.14) was significantly larger than that for the visual condition (0.31 ± 0.043, *p* = 0.0040) and the combined condition (0.32 ± 0.055, *p* = 0.017). The mean *σ* for the latter two conditions was not significantly different from each other (*p* = 1.00).

In summary, the results of this experiment show marked differences among the conditions regarding the extent to which the self-movement component is removed from the retinal motion of the probe for the perception of scene-relative object motion during walking. Although non-visual information alone supports about 50% and visual information (such as optic flow) alone supports about 80% removal of the self-movement component, combined visual and non-visual information supports close to 100% removal and thus yields almost perfect estimation of scene-relative object motion. Furthermore, although non-visual information is used for the perception of scene-relative object motion during walking, the precision of object motion estimation with non-visual information alone compared with visual information alone or combined visual and non-visual information is lowered by about a factor of 2. Taken together, these results indicate that visual information plays a more important role in the perception of scene-relative object motion during walking than does non-visual information, as indicated by the larger percentage compensation gain and higher precision in object motion estimation with visual than non-visual information. Nevertheless, neither visual nor non-visual information alone is sufficient for accurate perception of scene-relative object motion during self-movement. In a natural environment, both are available during walking, and our results indicate that it is the combination of both visual and non-visual information that enables us to accurately perceive scene-relative object motion during walking.

## Experiment 2: Does the perceived probe distance change in different scenes?

A possible alternative explanation of the results of Experiment 1 is that the availability of different depth cues in the three stimulus conditions affected the perceived distance of the probe and thus affected the perceived probe motion (Gogel, 1982; Gogel & Tietz, 1992). Specifically, the random-dot ground scene contained monocular depth cues that were not present in the empty ground scene; thus, observers might have perceived the probe at a closer distance in the random-dot than in the empty ground scene. Due to the fact that the perceived self-movement component in the retinal motion of the probe is larger when the probe is perceived at a closer distance and smaller when the probe is perceived at a farther distance (a phenomenon known as motion parallax), this could have led to a larger self-movement component being subtracted and thus a higher percentage compensation gain observed for the visual- and the combined-information conditions, which both used the random-dot ground scene, than for the non-visual-information condition, which used the empty ground scene. In this experiment, we examined this possibility.

### Methods

#### Participants

Twelve new participants (all naive as to the specific goals of the study; seven males, five females) between the ages of 24 and 32 years participated in this experiment. All had normal or corrected-to-normal vision and provided informed consent. The study was approved by the Institutional Review Board at the New York University Shanghai.

#### Visual stimuli and procedure

**Figure 3.**
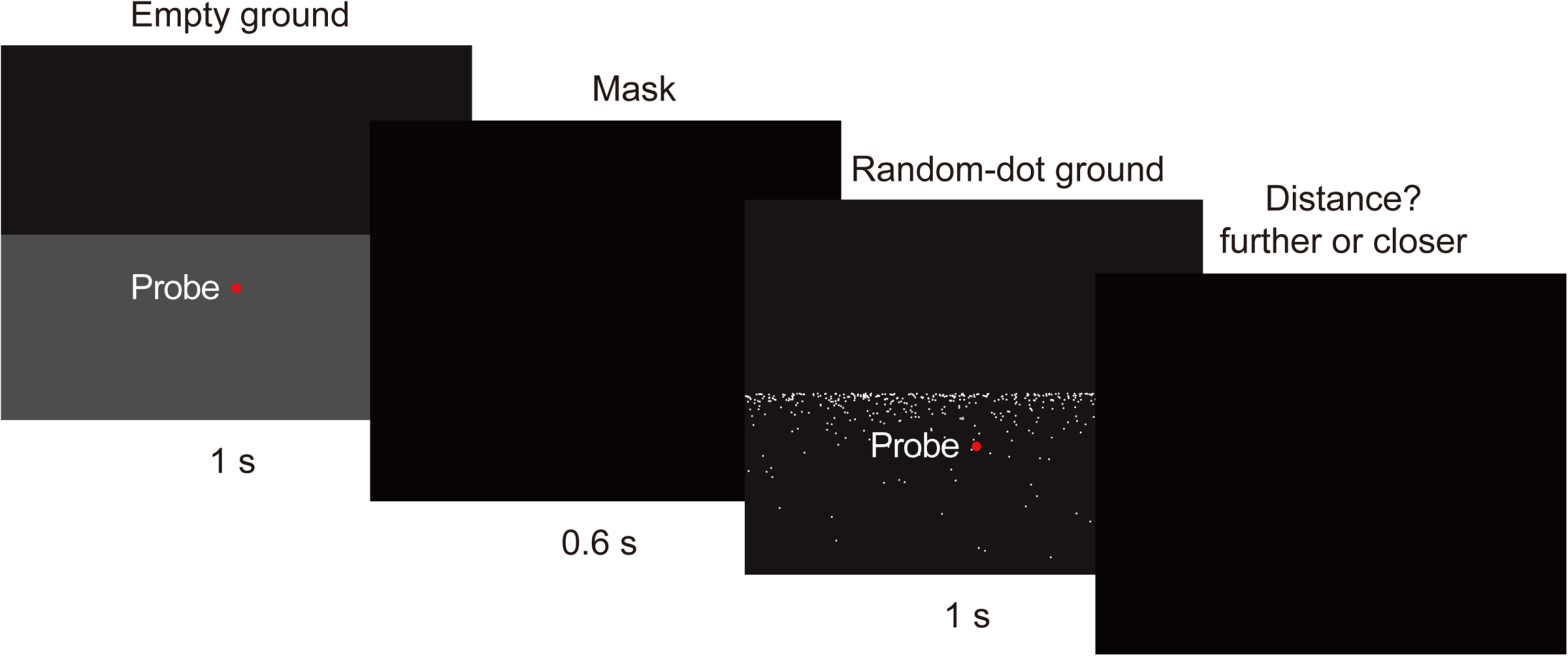
Illustration of Experiment 2 procedure. The presentation order of the empty ground versus the random-dot ground scene was randomly varied from trial to trial.

Participants viewed the same empty ground and the random-dot ground scenes of Experiment 1 through the HMD. We used a two-interval, forced-choice procedure to examine any difference in the perceived distance of the probe object in these two stimulus conditions (Figure 3). Specifically, on each trial, each participant first viewed one of the two scenes for 1 second. For the random-dot ground scene, the red probe dot (1° diameter) was positioned at 7.3 m in front of the participant, which was the distance of the midpoint of the trajectory of the probe in Experiment 1. For the empty ground scene, the distance of the probe dot was varied from trial to trial by the Bayesian adaptive method (Kontsevich & Tyler, 1999). After viewing the first scene, the participant was presented with a black screen for 0.6 seconds and then the second scene for 1 second, after which the screen turned black again. Trials were randomly intermixed. For half of the trials, the participant viewed the empty ground scene first; for the other half, the participant viewed the random-dot ground scene first. A random yaw rotation drawn from a uniform distribution between –5° and 5° was added to the second scene in each trial to prevent observers from performing the task by using two-dimensional image cues to find the probe location. At the end of each trial, the participant was asked to indicate whether the probe distance in the second scene was farther or closer than that in the first scene using a handheld controller.

As in Experiment 1, before the start of each trial, each participant went through a calibration procedure to make sure his/her body and head were oriented to face the *z*-axis of the virtual world. The calibration procedure was the same as in Experiment 1. After the participant held this position for 1 second, the trial started. Forty trials were run for the distance judgment task. Before the experiment started, the participant received five practice trials. This experiment lasted about 10 minutes.

#### Data analysis

The Bayesian adaptive method (Kontsevich & Tyler, 1999) allowed us to compute the PSE distance offset—that is, the distance that was added to the probe in the empty ground scene so that the perceived probe distance was the same in the two scenes. A positive PSE distance offset indicates that the probe in the empty ground scene has to be placed farther than in the random-dot ground scene for the perceived distance of the probe to be the same in the two scenes, which means the perceived distance of the probe is closer in the empty than the random-dot ground scene. In contrast, a negative PSE distance offset indicates that the probe in the empty ground scene has to be placed closer than in the random-dot ground scene for the perceived distance of the probe to be the same in the two scenes, which means that the perceived distance of the probe is closer in the random-dot than the empty ground scene.

### Results and discussion

**Figure 4.**
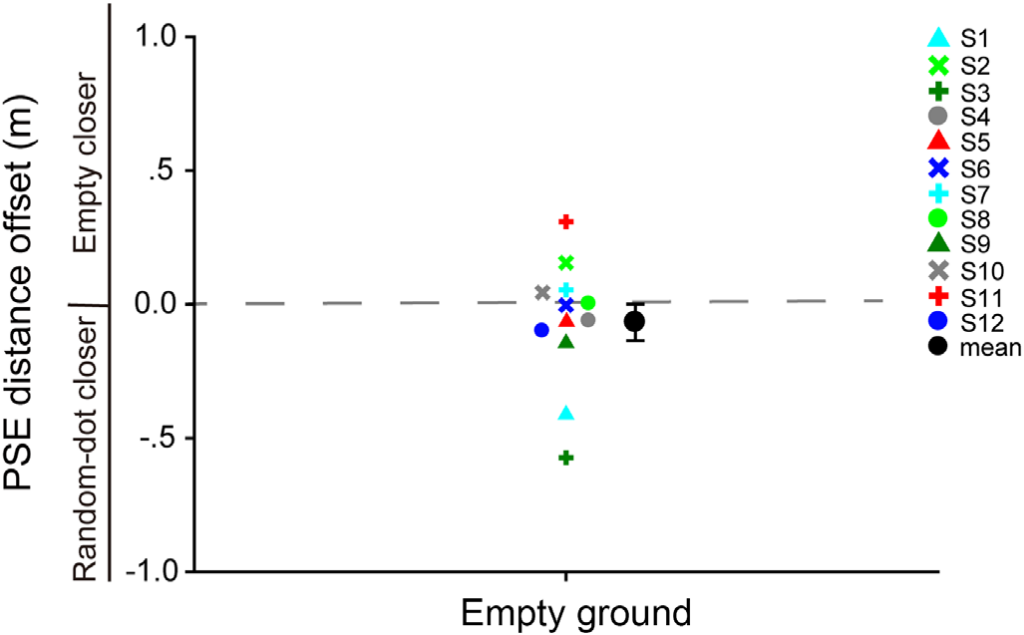
Experiment 2 data. PSE distance offset for each participant along with the mean. Error bars are ±1 *SE* across 12 participants.

Figure 4 plots the PSE distance offset for each participant along with the mean. A one-sample *t*-test revealed that the mean PSE distance offset (mean ± *SE*, –0.068 ± 0.068 m) was not significantly different from zero, *t*(11) = –0.99, *p* = 0.34, Cohen's *d* = 0.29. This indicates that the perceived probe distance was not significantly different across the empty ground and the random-dot ground scenes, despite the fact that the two scenes provided different depth cues. As such, it is unlikely that the results of Experiment 1 were due to any change in the perceived probe distance across different stimulus conditions. Indeed, salient depth cues such as binocular disparity signaling near depth were always available in both scenes, which could have contributed to the unchanging perception of the probe distance across different stimulus conditions.

## General discussion

Combining the results of the two experiments, we show that, during walking, non-visual information contributes to about 50% removal of the self-movement component in the retinal motion of the probe for the perception of scene-relative object motion, but visual information plays a more significant role in this process, as indicated by a larger proportion of self-motion component removed (about 80%) and more than two times higher precision in object motion estimates. However, only when both visual and non-visual information is available can observers accurately perceive the scene-relative object motion during walking. When only visual or non-visual information is available, the perception of scene-relative object motion is biased due to incomplete removal of the self-movement component from the retinal motion of the probe, and this bias is unlikely due to any misperception of object distance.

The approximately 50% self-movement compensation observed in our non-visual-information condition that involved active walking is consistent with the findings of two previous studies that asked observers to judge the speed of a moving object during passive and active head movement (Wexler, 2003) or body oscillation (Dyde & Harris, 2008) in the absence of visual information about self-movement. Specifically, these two studies reported that passive self-movement led to about 40% compensation of the self-movement component for the estimation of object speed, and this compensation increased to about 50% during active self-movement. In contrast, Dokka et al. (2015) did not observe any compensation for the self-movement component when judging object motion trajectory in their condition of vestibular information alone. This could be due to the fact that they only examined the vestibular information generated during passive leftward or right self-movement at low speeds (peak speed ≤ 32 cm/s). Together, these results indicate that non-visual information other than vestibular information as well as efference copy signals generated during active self-movement (Bridgeman, 1995; Wertheim, 1994) likely all play important roles in the removal of the self-movement component from the retinal motion of the probe for the accurate perception of scene-relative object motion.

In the current study, when self-movement was visually simulated in the visual-information condition, the percentage compensation gain increased to about 80%, similar to what was observed in our previous study that used the same nulling method but a much richer optic flow scene (Niehorster & Li, 2017). Note that other previous studies that quantitatively examined the contribution of visual information alone to the perception of scene-relative object motion during simulated self-movement reported about 40% to 60% compensation (Dokka et al., 2015; Dupin & Wexler, 2013). Given that the current study and these previous studies tested different self-movement speeds and object motion speeds, the difference in the observed percentage compensation gains could come from different self-movement or object motion speeds tested. However, this is unlikely due to the fact that our previous study found that flow parsing gain was relatively constant across common self-movement or object motion speeds experienced in daily life (Niehorster & Li, 2017). We surmise that the higher percentage compensation gain observed in our visual-information condition could be due to the fact that we tested a more frequently encountered form of self-movement in daily life (i.e., forward translation), using an immersive virtual reality experimental setup with a large field of view (100° diagonal field of view).

When both visual and non-visual information about self-movement was available in our combined-information condition, we observed an increased percentage compensation gain (98%) when judging object motion during walking compared with our non-visual and visual conditions. This is consistent with previous studies that also reported an increased compensation gain with combined visual and non-visual information compared to either visual information alone (Dokka et al., 2015) or non-visual information alone (Dyde & Harris, 2008). Dupin and Wexler (2013) also compared the gain of self-movement compensation in visual only, non-visual only, and combined conditions when judging the speed of object rotation. Even though visual and non-visual self-movement cues were placed in conflict in their combined condition, they still observed an increased compensation gain compared with their visual or non-visual only condition. In contrast to the current study and previous studies (Dupin & Wexler, 2013; Dyde & Harris, 2008) that have tested active self-movement reported >90% compensation with combined visual and non-visual information, Dokka et al. (2015) tested passive self-movement and reported a percentage compensation gain of about 60% with combined visual and vestibular information. This further supports the claim that the information generated during active self-movement (such as efference copy signals) makes an important contribution to the perception of scene-relative object motion during self-movement.

Regarding the precision of scene-relative object motion estimation during self-movement, we found that the precision was worse with non-visual information alone than with visual information alone or combined visual and non-visual information. The precision improved more than twofold with visual information alone but did not further improve with combined visual and non-visual information about self-movement. Only one previous study examined how the precision of judging scene-relative object motion changed with the availability of different self-movement cues and reported that the precision was better with visual information alone but decreased with added vestibular information (Dokka et al., 2015). The difference could be due to the fact that head bobbing and body sway generated during active walking in the current study produced additional vestibular and visual information compared to what was provided by passive self-movement on a sled in Dokka et al. (2015). For example, although bounce and sway of the head and body during active walking added visual jitter/oscillation noise to the scene, it has been reported that such visual jitter/oscillation noise could enhance the perception of vection and travel distance (e.g., Bossard, Goulon, & Mestre, 2016; Kim & Palmisano, 2008; Palmisano, Gillam, & Blackburn, 2000). In addition, apart from the fact that efference copy signals were absent during passive self-movement, non-visual cues generated during active walking contain not only vestibular information but also somatosensory and proprioceptive information about self-movement. All of these factors could have led to the improved precision in scene-relative object motion estimation in the combined-information condition in the current study.

In two studies using a similar virtual reality setup with an HMD, Fajen and his colleagues examined the contribution of visual and non-visual information about self-movement to judgments of obstacle avoidance; that is, participants judged whether they would pass in front of or behind a cylinder object that moved from right to left (Fajen & Matthis, 2013) during forward walking or whether they could safely pass through the gap between two converging cylinders in a virtual environment (Fajen et al., 2013). They found that both visual and non-visual information about self-movement affected such judgments and proposed that in real, actively generated self-movement such as walking both types of information contribute to the identification of scene-relative object motion. However, due to the design of these two studies, they did not address the questions of whether and how using visual and non-visual information about self-movement improves the accuracy with which people perceive scene-relative object motion during self-movement, which were directly answered by our current study.

Regarding the underlying mechanism accounting for different compensation gains observed with different sources of information about self-movement, it could be due to the variation in the accuracy of the estimation of self-movement with different self-movement cues. It has been reported that non-visual information (such as vestibular signals, proprioception, and somatosensory information) contributes to the estimation of self-movement (Crowell, Banks, Shenoy, & Andersen, 1998), but visual information such as optic flow helps the estimation of self-movement based implicitly on an assumption of visual stationarity (for a review, see Lappe, Bremmer, & van den Berg, 1999). As such, previous studies that asked observers to estimate self-movement speed from expanding optic flow stimuli viewed through an HMD found that the estimated speed was reduced by active physical translation such as walking on a treadmill (Banton, Stefanucci, Durgin, Fass, & Proffitt, 2005; Durgin, Gigone, & Scott, 2005; Thurrell & Pelah, 2005). That is, when optic flow is not synced to non-visual information generated during active walking on a treadmill, people tend to underestimate the speed of self-movement. Nevertheless, it still remains in question whether people perceive the speed of their self-movement more accurately with synched visual and non-visual information during real walking over the ground, which is different from walking on a treadmill. When walking over the ground, vestibular stimulation is also matched to proprioceptive and efference copy signals, which is not the case during walking on a treadmill, in which case the forward self-movement component is missing. The findings of the current study indicate the roles that visual and non-visual information play in the perception of scene-relative object motion during walking, but how they interact with each other for the accurate estimation of self-movement remains largely unknown and offers interesting perspectives for future research.

In summary, accurate perception and estimation of scene-relative object motion during self-movement is important for visual navigation. Using a motion-nulling procedure, we directly and quantitatively measured the self-movement component in object retinal motion that could be removed by relying on different sources of information about self-movement generated during walking. We found that neither visual nor non-visual information alone is sufficient for accurate scene-relative object motion judgments during self-movement. The availability of both is necessary for the accurate estimation of scene-relative object motion during walking. We thus conclude that accurate perception of scene-relative object motion during walking requires the integration of both visual and non-visual information about self-movement.

## Supplementary Material

Supplement 1

Supplement 2

Supplement 3

## Supplementary material

**Supplementary Movie S1**. Recorded video for the visual-information condition. The movie shows the scene viewed through the HMD that simulated smooth linear forward walking over the random-dot ground (i.e., with no head bobbing and body sway).

**Supplementary Movie S2**. Recorded video for the non-visual-information condition. The movie shows the scene viewed through the HMD during walking that depicted walking straight over the empty ground (i.e., with natural head bobbing and body sway generated during walking).

**Supplementary Movie S3**. Recorded video for the combined-information condition. The movie shows the scene viewed through the HMD during walking that depicted walking straight over the random-dot ground (i.e., with natural head bobbing and body sway generated during walking).
